# Nicotinamide riboside has minimal impact on energy metabolism in mouse models of mild obesity

**DOI:** 10.1530/JOE-21-0123

**Published:** 2021-08-09

**Authors:** David M Cartwright, Lucy A Oakey, Rachel S Fletcher, Craig L Doig, Silke Heising, Dean P Larner, Daniela Nasteska, Caitlin E Berry, Sam R Heaselgrave, Christian Ludwig, David J Hodson, Gareth G Lavery, Antje Garten

**Affiliations:** 1 Institute of Metabolism and Systems Research, College of Medical and Dental Sciences, University of Birmingham, Birmingham, UK; 2 School of Science and Technology, Nottingham Trent University, Nottingham, UK; 3 Pediatric Research Center, Hospital for Child and Adolescent Medicine, Leipzig University, Leipzig, Germany

**Keywords:** vitamin B3, NAD, C57BL/6J, high-fat diet, impaired glucose tolerance, mitochondrial function, mouse strain, energy expenditure

## Abstract

Supplementation with precursors of NAD has been shown to prevent and reverse insulin resistance, mitochondrial dysfunction, and liver damage in mouse models of diet-induced obesity. We asked whether the beneficial effects of supplementation with the NAD precursor nicotinamide riboside (NR) are dependent on mouse strain. We compared the effects of NR supplementation on whole-body energy metabolism and mitochondrial function in mildly obese C57BL/6N and C57BL/6J mice, two commonly used strains to investigate metabolism. Male C57BL/6N and C57BL/6J mice were fed a high-fat diet (HFD) or standard chow with or without NR supplementation for 8 weeks. Body and organ weights, glucose tolerance, and metabolic parameters as well as mitochondrial O_2_ flux in liver and muscle fibers were assessed. We found that NR supplementation had no influence on body or organ weight, glucose metabolism or hepatic lipid accumulation, energy expenditure, or metabolic flexibility but increased mitochondrial respiration in soleus muscle in both mouse strains. Strain-dependent differences were detected for body and fat depot weight, fasting blood glucose, hepatic lipid accumulation, and energy expenditure. We conclude that, in mild obesity, NR supplementation does not alter metabolic phenotype in two commonly used laboratory mouse strains.

## Introduction

Repletion of NAD with vitamin B3 precursors or metabolites, mainly nicotinamide riboside (NR) and nicotinamide mononucleotide (NMN) has been used widely in experimental animal studies to counteract negative consequences of metabolic and mitochondrial disorders. Beneficial effects on energy metabolism and mitochondrial function were found in animal models of diet-induced obesity (Yoshino *et al.* 2011, Cantó *et al.* 2012, Yoon *et al.* 2015, Mills *et al.* 2016, de Castro *et al.* 2020), liver damage (Gariani *et al.* 2016, Zhou *et al.* 2016, Mukherjee *et al.* 2017, Wang *et al.* 2018, Han *et al.* 2019), diabetes (Kakimoto & Kowaltowkski 2016, Stromsdorfer *et al.* 2016, Trammell *et al.* 2016), and mitochondrial disease (Khan *et al.* 2014, Schöndorf *et al.* 2018). Several of these studies (Cantó *et al.* 2012, Gariani *et al.* 2016, Trammell *et al.* 2016, Zhang *et al.* 2016, Zhou *et al.* 2016, Wang *et al.* 2018, Han *et al.* 2019) were conducted with male C57BL/6J or C57BL/6JRj mice (B6J), a commonly used mouse model to study the effects of diet-induced obesity and glucose intolerance. A limitation of using these mice for metabolic studies is a deficiency in nicotinamide nucleotide transhydrogenase (Nnt), a mitochondrial enzyme crucial for maintaining redox balance (Toye *et al.* 2005, Ronchi *et al.* 2013). Mitochondria of B6J mice have a lower peroxide removal capacity as well as increased sensitivity to Ca2+-mediated permeability transition (Ronchi *et al.* 2013, 2016, Lopert & Patel 2014, Navarro *et al.* 2017). Studies assessing the effects of non-functional Nnt in mice found derangements in glucose homeostasis and aggravated fatty liver disease (Toye *et al.* 2005, Williams *et al.* 2014, Fisher-Wellman *et al.* 2015, 2016, Rendina-Ruedy *et al.* 2015, Navarro *et al.* 2017) in B6J mice, although others did not see a difference in glucose metabolism (Wong *et al.* 2010).

Few human clinical trials have addressed supplementation of NAD with NR in elderly and mildly obese, but otherwise healthy, subjects. These studies reported no effects of NR supplementation on mitochondrial and endocrine pancreatic function as well as whole-body energy metabolism, although changes in muscle gene expression and circulating anti-inflammatory cytokines were detected (Dollerup *et al.* 2018, Martens *et al.* 2018, Dollerup *et al.* 2019*a*,*b*, Elhassan *et al.* 2019). The observed changes were accompanied by increased muscle nicotinic acid adenine dinucleotide, N-methyl nicotinamide, and N1-methyl-2 pyridone-5-carboxamide, while NAD^+^ was elevated in the blood, but not in muscle (Elhassan *et al.* 2019).

We asked whether the choice of mouse strain could affect outcomes of NAD augmentation studies. We compared the effects of NR supplementation in male C57BL/6J mice to C57BL/6N mice which are competent for Nnt (Fisher-Wellman *et al.* 2016). A mild obesity phenotype was induced by a diet high in saturated fat (60%) for 8 weeks (HFD), and whole-body energy metabolism and mitochondrial function were assessed. We found slight effects of NR on mitochondrial respiration in soleus muscle in both mouse strains that were not translated into decreases in body weight, improved glucose tolerance, or alterations in energy metabolism in either mouse strain. We conclude that a mild obesity phenotype is not influenced by NR supplementation in both B6N and B6J mice.

## Materials and methods

### Animals

Mice were purchased from Charles River, UK (B6J) and MRC Harwell, UK (B6N Taconic). Mice were group-housed in a standard temperature (22°C) and humidity-controlled environment with 12 h light:12 h darkness cycle, except for indirect calorimetry experiments, where animals were single-housed for 72 h. Nesting material was provided and mice had *ad libitum* access to food and water unless otherwise noted. Starting from an age of 7 weeks (9 weeks in case of B6N chow-fed mice), mice were fed either a standard chow (EUrodent diet 14%, 5LF2*, 0.0074% niacin) or a high-fat diet (HFD, Brogaarden D12492i, 60% calories from fat, 0.3% nicotinic acid) for 8 weeks. For NR supplementation, 3.0 mg/mL NR chloride (Chromadex Inc.) was dissolved in drinking water in light-protected bottles and changed every 3 days since this regimen was shown to lead to a stable increase in hepatic NAD (Mukherjee *et al.* 2017). NR stability was checked by incubation of NR in mouse drinking water at room temperature for 6 days followed by NMR quantification. After 6 days, the portion of NR was 95% (Supplementary Fig. 1A, see section on supplementary materials given at the end of this article). The effect of NR supplementation on total NAD levels in the liver and cardiac muscle of HFD-fed B6J mice was checked by an enzymatic cycling assay (NAD/NADH Quantitation Colorimetric Kit, BioVision, Inc.). We detected higher NAD levels both in the liver (Supplementary Fig. 1B) and cardiac muscle (Supplementary Fig. 1C) of NR-supplemented compared to non-supplemented mice.

Mice were weighed weekly. Mice were sacrificed using cervical dislocation after exsanguination under isofluorane anesthesia. Liver, inguinal, and epididymal fat as well as skeletal muscle (tibialis anterior (TA), soleus) were immediately frozen in liquid N_2_ or used for mitochondrial respiration measurements. Genotyping was performed according to Nicholson *et al.* (2010) for each mouse to verify grouping into B6N or B6J strain (Supplementary Fig. 1D). A subgroup of tissues was weighed. All animal experiments were conducted in accordance with UK Home Office regulations, UK Animals (Scientific Procedures) Act 1986 under project license number 70/8516.

### Glucose homeostasis assessment

Experiments were performed following a 5 h fast starting at ~08:00 h. Blood glucose was monitored using the Contour XT glucometer (Bayer) from samples collected at the distal tail vein. Following an initial blood glucose measurement, glucose (2 g/kg body weight) was injected intraperitoneally. Blood glucose was measured after 15, 30, 60, 90, and 120 min.

### Hepatic lipid accumulation

Quantification of liver triglycerides was performed using a triglyceride quantification kit (ab65336, Abcam) according to the manufacturer’s instructions. One lobe (lobus hepatis sinister) was fixed in 4% paraformaldehyde and paraffin-embedded. Sections were stained with hematoxylin and images were taken at 20× magnification.

### Indirect calorimetry

Energy expenditure, respiratory exchange ratio (RER), food and water intake of HFD-fed (*n = *10 for each group) and chow-fed (*n = *4 four for each group) were measured in a Phenomaster System (TSE systems, Bad Homburg, Germany). Briefly, animals were group-acclimatized for 24 h before isolation. On day 2 at 12:00 h, mice were removed from the home cages, weighed and their overall condition was checked, then placed into new cages in isolation. On the morning of day 3, mice were visually checked for signs of stress at 09:00 h and food/drink consumption was recorded. If mice were not eating and/or drinking (determined as less than 1 g of food or 1 mL of water), they were removed from the experiment (*n = *1 B6N mouse). For the data collection phase, mice were then left undisturbed until 13:00 h on day 5, where they were removed. Food/drink consumption data were checked again and mice were weighed. If the mice had not eaten or drank and/or had lost more than 20% body weight since day 1, the data were excluded from the experiment (*n = *2 B6N mice). The last 48 h were used for data analysis. ∆RER was calculated by subtracting the mean of the five highest RER values during the dark phase from the mean of the five lowest RER values during the light phase. Caloric consumption was calculated using the following values: HFD 5.24 kcal/g, chow 3.18 kcal/g.

### Assessment of mitochondrial function

Mitochondrial respiration was measured by high-resolution respirometry (Oxygraph2k, Oroboros, Austria). Liver mitochondria were isolated according to Frezza *et al.* (2007). Mitochondrial protein concentration was measured using the Bio-Rad DC protein assay. Permeabilization of skeletal muscle fibers was performed according to Pesta & Gnaiger (2012). After weighing, muscle fibers were placed in the Oxygraph2k chamber and O2 concentration was raised to approx. 400 μM. Substrates, uncoupler, and inhibitors were added to the chambers in the following sequence: malate (0.05 mM), octanoyl-carnitine (0.2 mM), ADP (4 mM final concentration), malate (2.05 mM final concentration), glutamate (10 mM), succinate (20 mM), carbonyl cyanide-4-(trifluoromethoxy) phenylhydrazone (FCCP, sequential addition of 0.25 µM until no further increase in O2 consumption), rotenone (0.5 µM), and antimycin A (2.5 µM) to obtain values for residual oxygen consumption (ROX). O_2_ flux values were normalized after subtraction of ROX values to wet weight (muscle fibers) or protein concentration (liver mitochondria).

### Citrate synthase assay

Citrate synthase (CS) activity in liver mitochondria and homogenized muscle samples was measured in a microplate format according to Horscroft *et al.* (2017). Briefly, samples were either homogenized (muscle fibers) or diluted (liver mitochondria) in CS sample buffer (Hepes 20 mM, EDTA 1 mM, Triton-X100 0.1% (v/v), protein concentration was measured (Bio-Rad DC protein assay) and adjusted to 10 μg/mL in assay buffer (Tris 100 mM, pH 8.0, 5,5’-Dithiobis-(2-nitrobenzoic acid) (DTNB) 0.1 mM, acetyl coenzyme A (CoA) 0.3 mM). To start the reaction, oxaloacetate was added to a final concentration of 0.5 mM and the reaction of DTNB with free CoA-SH was monitored spectrophotometrically at 412 nm (Horscroft *et al.* 2017).

### Statistical analyses

**Figure 1:**
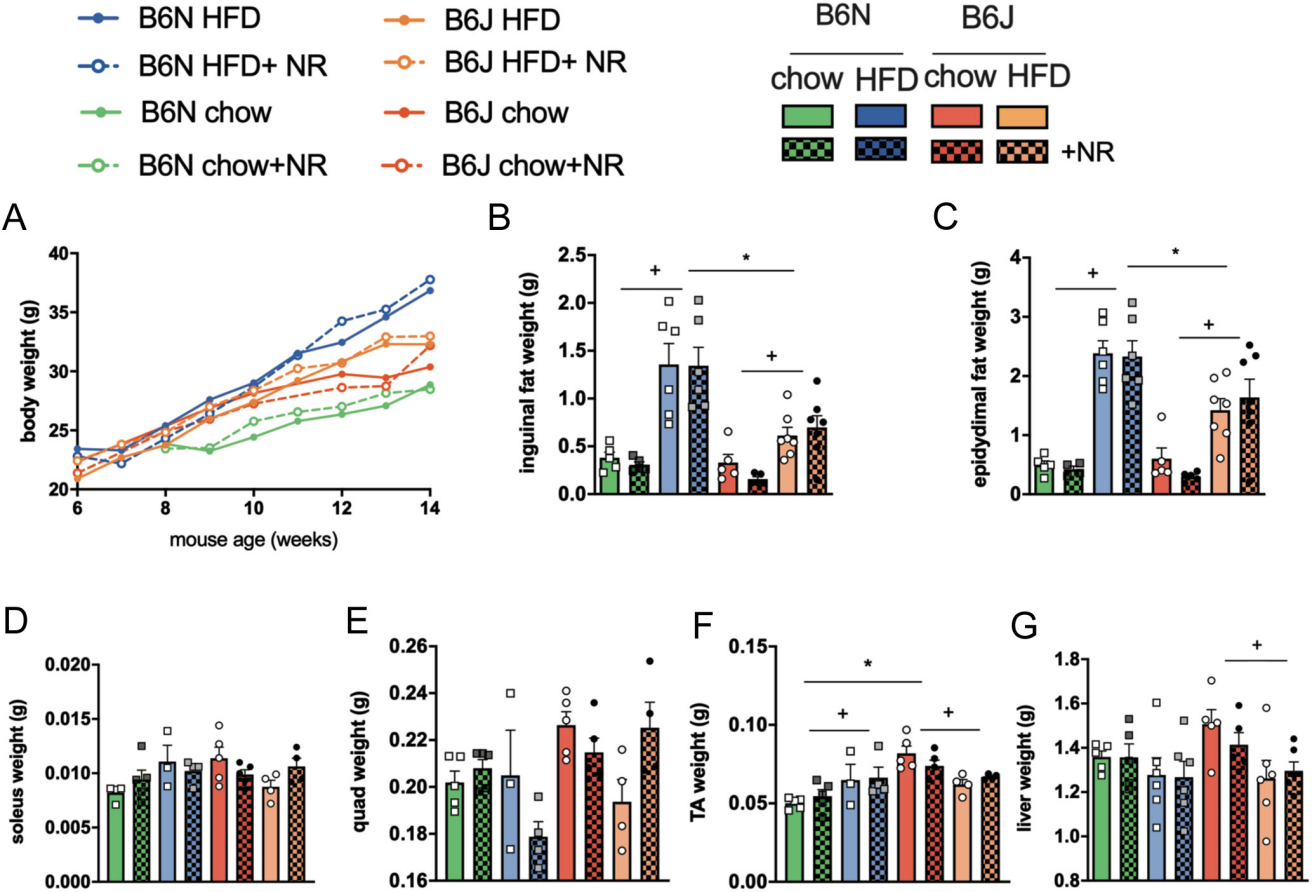
NR supplementation does not counteract HFD-induced obesity. (A) Bodyweight during 8 weeks of chow or HFD feeding of B6N and B6J mice, (B) inguinal, (C) epididymal fat pad, (D) liver, (E) quadriceps, (F) tibialis anterior, and (G) soleus muscle weight. Data are presented as mean ± s.e.m., *n = *10 HFD, *n*  =5 chow for body weight, *n = *6 HFD, *n = *5 chow for liver and fat weights, *n = *3–5 for muscle weights. Tests for statistical significance were done using repeated three-way ANOVA, with time, diet, and mouse strain as factors (A) or ordinary two-way ANOVA with diet and mouse strain as factors followed by Tukey’s test for multiple comparisons. Data for NR vs no NR supplementation were analyzed together since no significant differences were found for NR vs no NR. ^+^*P  <* 0.05 for HFD vs chow, ^*^P  < 0.05 for B6J vs B6N.

Strategy: our main aim was to check whether NR supplementation has a differential effect on B6J and B6N mice during HFD feeding. We first analyzed the effect of NR supplementation with supplementation (NR vs no NR) and mouse strain (B6J vs B6N) as factors in HFD-fed and chow-fed mice separately. Since we unexpectedly saw no effect of NR supplementation, but a difference in response to HFD feeding between mouse strains, we then tested for differences using mouse strain (B6J vs B6N) and diet (HFD vs chow) as factors and included mouse data regardless of whether or not mice were supplemented with NR. Tests for statistical significance were done using GraphPad Prism version 9 (GraphPad Software, LLC) with ordinary two-way ANOVA followed by Tukey’s multiple comparisons test, except for the time course of body weight data (Fig. 1A), which were analyzed by repeated three-way ANOVA with time, diet, and mouse strain as factors and for mitochondrial function data, which were analyzed by ordinary two-way ANOVA followed by Tukey’s multiple comparisons test, with mouse strain and NR supplementation as factors.

Multiple *t*-tests with Holm–Sidak correction were used for analyzing differences in time courses for energy expenditure and respiratory exchange ratio (RER). Energy expenditure and ∆RER were analyzed using linear regression and analysis of covariance (ANCOVA) between average absolute daily energy expenditure/∆RER and body weight (Mina *et al.* 2018). Differences between the slopes of the lines were determined by linear regression. All data points were included in the analysis. Data are represented as mean ± s.e.m., statistically significant (*P*  < 0.05) differences were marked by ^+^ for comparisons between chow and HFD, by ^*^ for comparisons between B6J and B6N mouse strains, and by ^#^ for comparisons between NR vs no NR supplementation.

## Results

### Body composition, glucose tolerance, and hepatic lipid accumulation were not altered by NR supplementation

Previous studies reported that NR supplementation counteracted negative effects of HFD-induced obesity, decreased fat depot size in HFD-fed mice (Cantó *et al.* 2012), and prevented HFD-induced impairments in glucose tolerance (Yoshino *et al.* 2011, Cantó *et al.* 2012, Trammell *et al.* 2016). We examined the impact of supplementation with NR on body and organ weight, fat depot weight, glucose tolerance, and lipid accumulation in the liver of B6N mice in comparison to B6J mice when fed standard chow and after HFD feeding. NR supplementation neither altered weight gain (Fig. 1A) nor affected the weight of adipose tissue depots, liver, and different muscles (Fig. 1B, C, D, E, F and G). In contrast, marked differences were detected in body weight gain of HFD-fed B6J vs B6N (Supplementary Fig. 1E). While B6N mice on HFD had a significantly higher body weight than B6N chow-fed mice starting from week 9, B6J mice on HFD differed from chow-fed B6J only in week 13. There was a significant difference in final weight (week 14) of HFD-fed B6N and B6J mice, while chow-fed B6N and B6J mice did not differ significantly. Fat depot weights of HFD-fed mice were higher than chow-fed mice, while HFD-fed B6N had a higher increase in fat depot weight than B6J mice (Fig. 1B and C). These differences persisted also when weights were normalized to body weight (Supplementary Fig. 1F and G). Soleus and quadriceps muscle were not significantly different in weight (Fig. 1D and E), while tibialis anterior muscle weight was higher in chow than in HFD-fed mice and in B6J vs B6N chow-fed mice (Fig. 1F, ^+^*P*  < 0.05 B6J HFD vs chow-fed mice, ^*^P  < 0.05 chow-fed B6J vs B6N). The liver weight of B6J chow-fed mice was higher than B6J HFD-fed mice (Fig. 1G, ^+^*P*  < 0.05 B6J HFD vs chow-fed mice).

**Figure 2:**
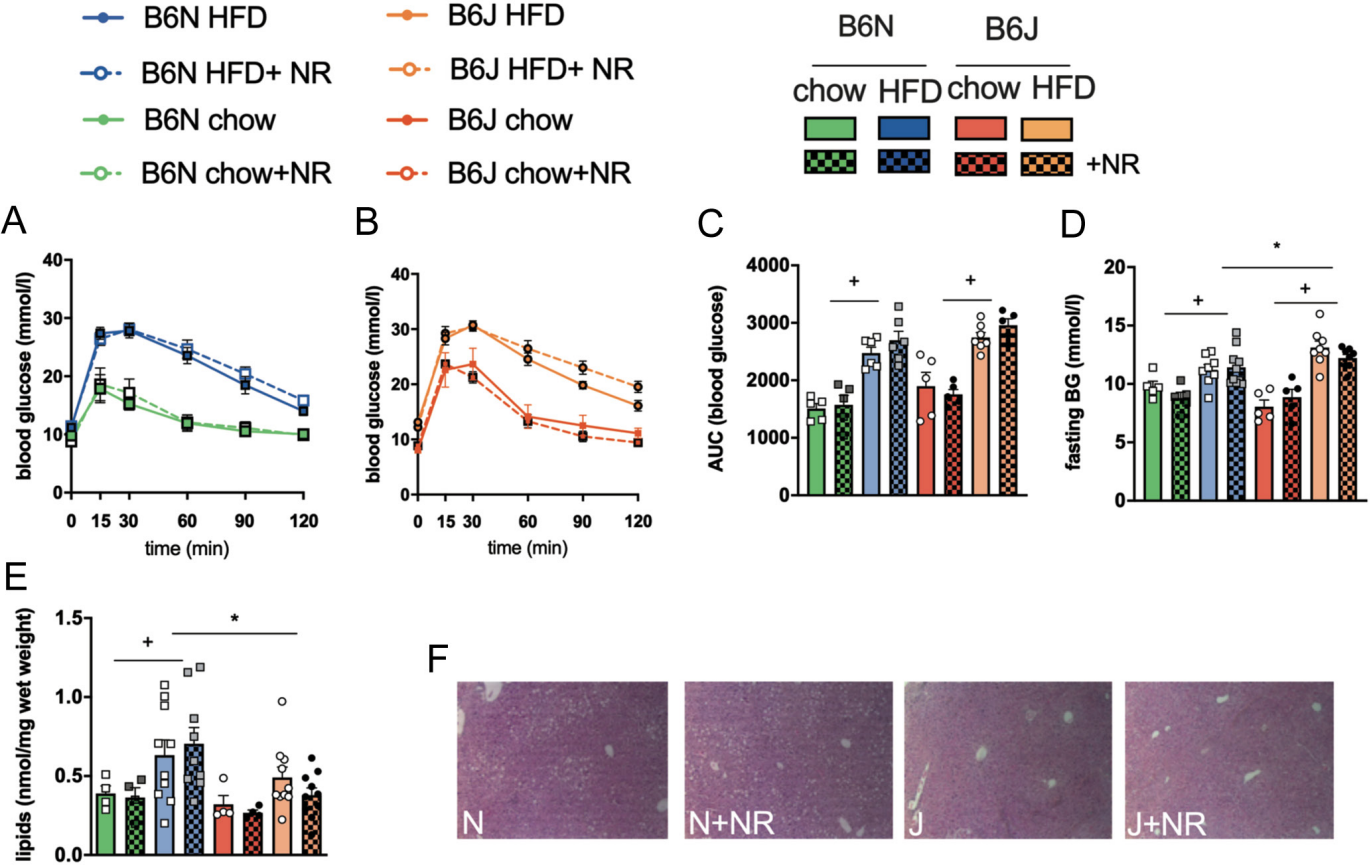
NR supplementation does not counteract HFD-induced glucose intolerance. Glucose tolerance after 8 weeks of chow or HFD feeding was assessed by i.p. glucose tolerance test with 2 g glucose/kg body weight after 5 h of fasting. Time course of blood glucose concentrations is shown in (A) B6N and (B) B6J mice and quantified in (C) area under the curve (AUC) *n = *6–7 HFD, *n* = 5 chow, (D) fasting blood glucose (BG) *n = *8–10 HFD, *n = *5 chow. (E) Hepatic lipid accumulation after 8 weeks of HFD or chow feeding, (F) representative images of H&E stained liver tissue sections (magnification 20×) of HFD-fed B6N and J mice. Data are presented as mean ± s.e.m., *n = *9–10 HFD, *n* = 4 chow. Tests for statistical significance were done using ordinary two-way ANOVA with diet and mouse strain as factors followed by Tukey’s test for multiple comparisons. Data for NR vs no NR supplementation were analyzed together since no significant differences were found for NR vs no NR. ^+^*P  <* 0.05 for HFD vs chow, *P  < 0.05 for B6J vs B6N.

HFD feeding for 8 weeks (Fig. 2A, B and C) induced a marked glucose intolerance both in B6N and B6J mice regardless of NR supplementation compared to chow-fed mice as measured by area under the curve (Fig. 2C, ^+^*P*  < 0.05 HFD vs chow-fed mice). After 8 weeks, HFD-fed mice of both strains had higher fasting blood glucose levels than mice on chow feed, while HFD-fed B6J mice displayed increased fasting blood glucose levels compared to HFD-fed B6N (Fig. 2D, ^+^*P*  < 0.05 HFD vs chow-fed mice, ^*^P  < 0.05 HFD-fed B6J vs B6N).

HFD-induced obesity is associated with increased hepatic lipid accumulation. NR supplementation did not decrease lipid accumulation in any experimental group. B6N mice on HFD stored significantly more hepatic lipids than chow-fed B6N mice or HFD-fed B6J, while there was no significant difference between HFD and chow-fed B6J mice (Fig. 2E and F, ^+^*P*  < 0.05 B6N HFD vs chow; ^*^P  < 0.05 HFD-fed B6J vs B6N).

### Energy metabolism is not influenced by NR supplementation

**Figure 3:**
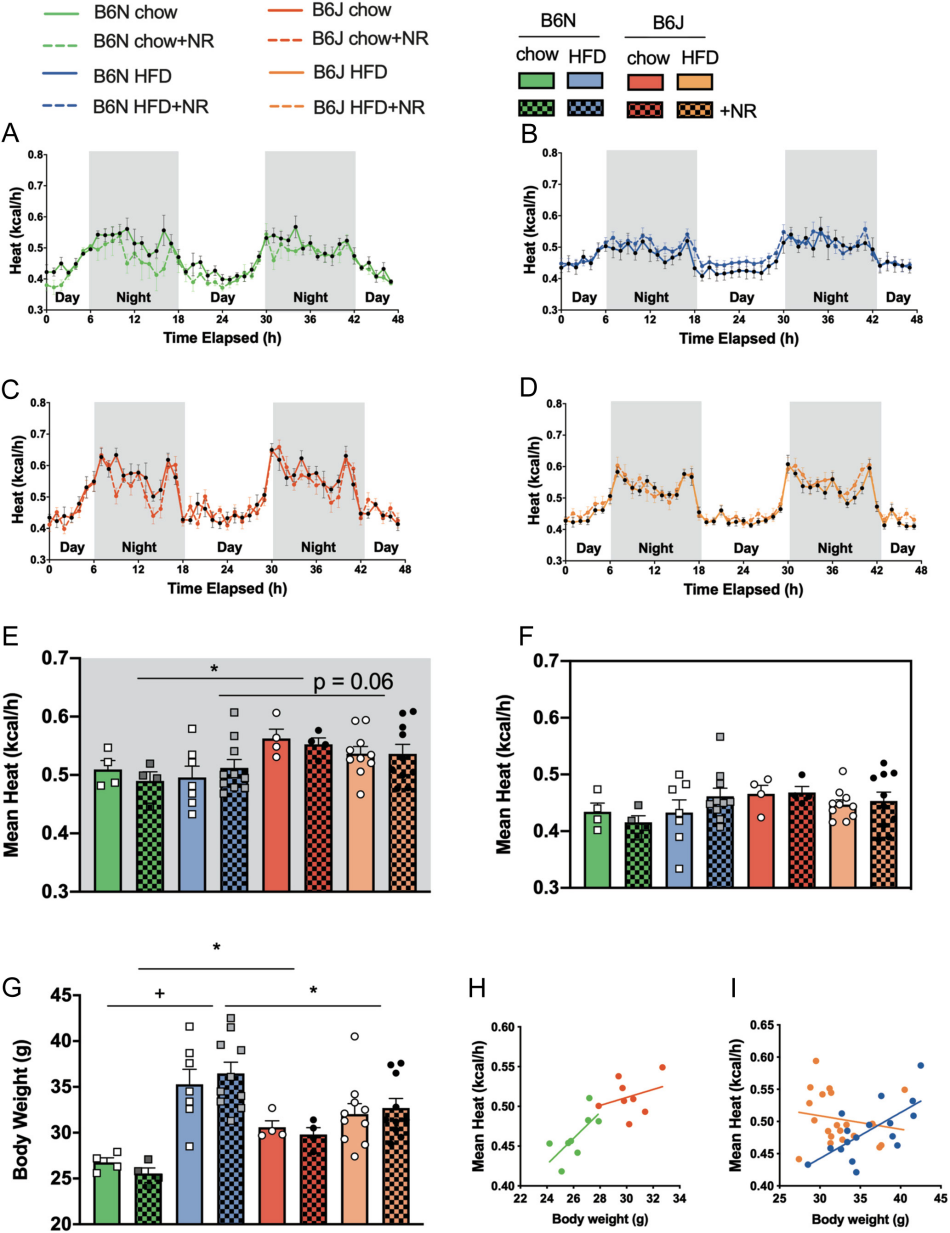
NR supplementation does not affect energy expenditure in B6N or B6J mice. Energy expenditure was determined by indirect calorimetry in metabolic cages. The time course of the energy expenditure measurement is shown in (A) chow-fed B6N, (B) chow-fed B6J, (C) HFD-fed B6N, (D) HFD-fed B6J. Differences were tested by multiple *t*-tests with Holm–Sidak correction. Differences in average energy expenditure between chow-fed and HFD-fed B6N and B6J mice are shown for (E) the active phase/night and (F) the resting phase/day, body weights at the start of indirect calorimetry assessment are shown in (G) (*n = *4 for chow, *n = *7 for B6N HFD, *n = *10 for B6N HFD+NR, B6J HFD and HFD+NR). The relationship between average nightly energy expenditure and body weight is depicted for B6J and B6N mice when chow-fed (H) and (I) HFD (*n = *8 for chow, *n = *17 for B6N HFD, *n = *20 for B6J HFD). Data are presented as mean ± s.e.m. Tests for statistical significance were done using ordinary two-way ANOVA with diet and mouse strain as factors followed by Tukey’s test for multiple comparisons. Data for NR vs no NR supplementation were analyzed together since no significant differences were found for NR vs no NR. ^+^*P  <* 0.05 for HFD vs chow, ^*^P  < 0.05 for B6J vs B6N Linear regression and analysis of covariance (ANCOVA) was done between average nightly energy expenditure and body weight. Slopes of HFD-fed mice were significantly different (*P  <* 0.05).

NR supplementation has been shown to enhance oxidative metabolism and energy expenditure in HFD-fed mice (Cantó *et al.* 2012). To test whether NR supplementation influenced the metabolic phenotype in our experimental setup, mice underwent indirect calorimetry measurements after 8 weeks of NR-supplemented or non-supplemented chow or HFD feeding. NR supplementation did not significantly affect energy expenditure in B6N or B6J mice regardless of diet (Fig. 3A, B, C and D). During the active phase, we observed a higher energy expenditure in B6J compared to B6N mice (*P*  < 0.05 for chow-fed mice; *P*  = 0.06 for HFD-fed mice). Chow-fed B6J mice had higher body weights than B6N on the chow diet as opposed to HFD-fed B6J mice, which had lower body weights than HFD-fed B6N mice (Fig. 3G). Interaction analysis showed a significant influence of body weight on energy expenditure in HFD-fed (Fig. 3F), but not chow-fed mice (Fig. 3E).

**Figure 4:**
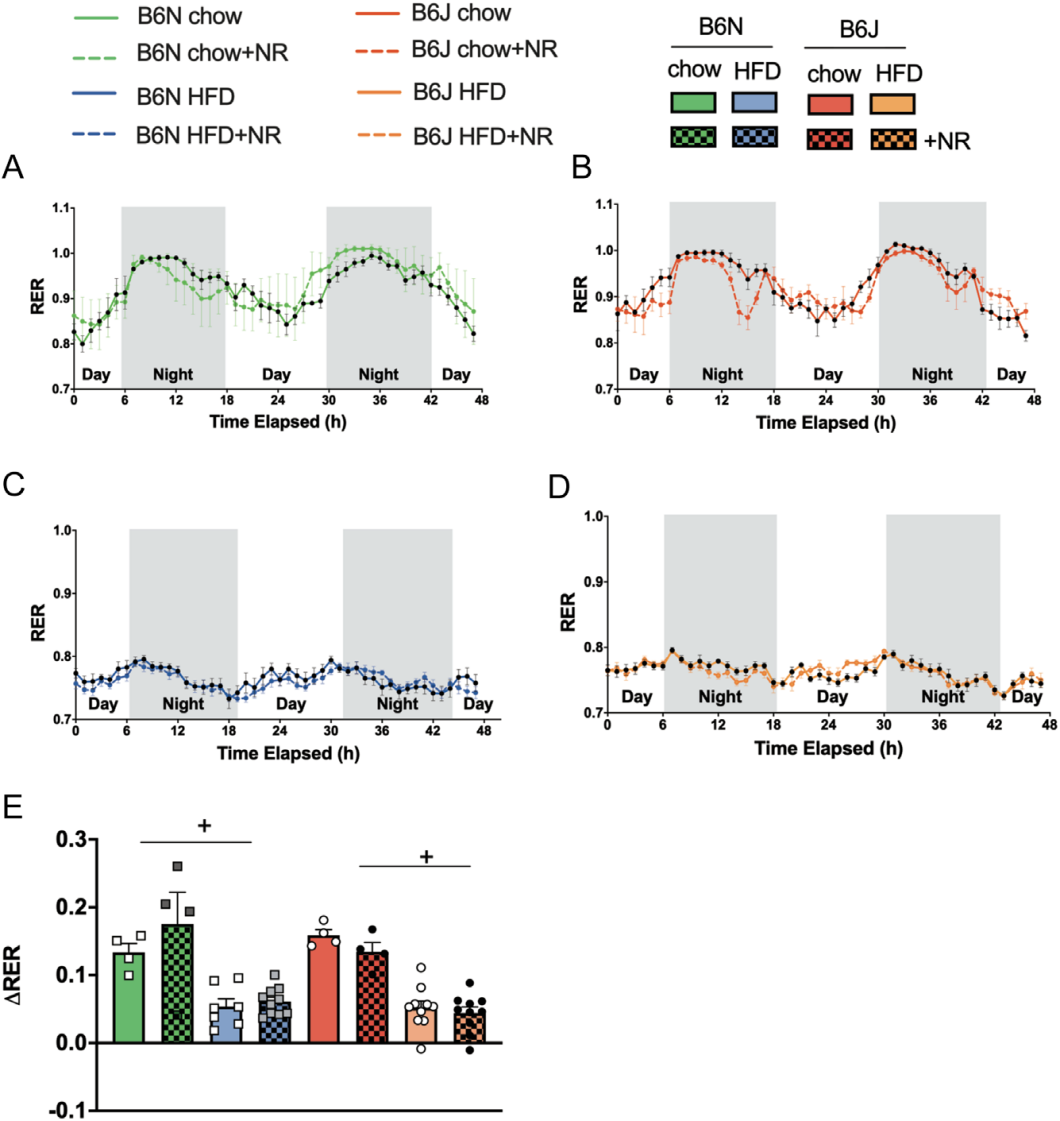
Substrate usage is not altered by NR supplementation. The respiratory exchange ratio (RER) as a measure for substrate usage was determined by indirect calorimetry in metabolic cages. The time course of RER measurement is shown in (A) chow-fed B6N, (B) chow-fed B6J, (C) HFD-fed B6N, and (D) HFD-fed B6J mice. ΔRER as a measure for metabolic flexibility was calculated by subtracting the five lowest values during the day from the five highest values during the night and is shown in (E). Data are presented as mean ± s.e.m., *n = *4 for chow, *n = *7 for B6N HFD, *n = *10 for B6N HFD+NR, B6J HFD, and HFD+NR. Tests for statistical significance were done using ordinary two-way ANOVA with diet and mouse strain as factors followed by Tukey’s test for multiple comparisons. Data for NR vs no NR supplementation were analyzed together since no significant differences were found for NR vs no NR. ^+^*P  <* 0.05 for HFD vs chow.

NR supplementation did not influence the respiratory exchange ratio (RER). The RER was higher in chow-fed than HFD-fed mice and indicated a preferential use of lipids after 8 weeks of HFD (Fig. 4A, B, C and D). As expected, metabolic flexibility as measured by the difference of RER between night and day (ΔRER) was higher in chow-fed than HFD-fed mice (Fig. 4E) and not influenced by mouse strain. Bodyweight did not influence ΔRER, as shown by ANCOVA (Supplementary Fig. 2A and B).

**Figure 5:**
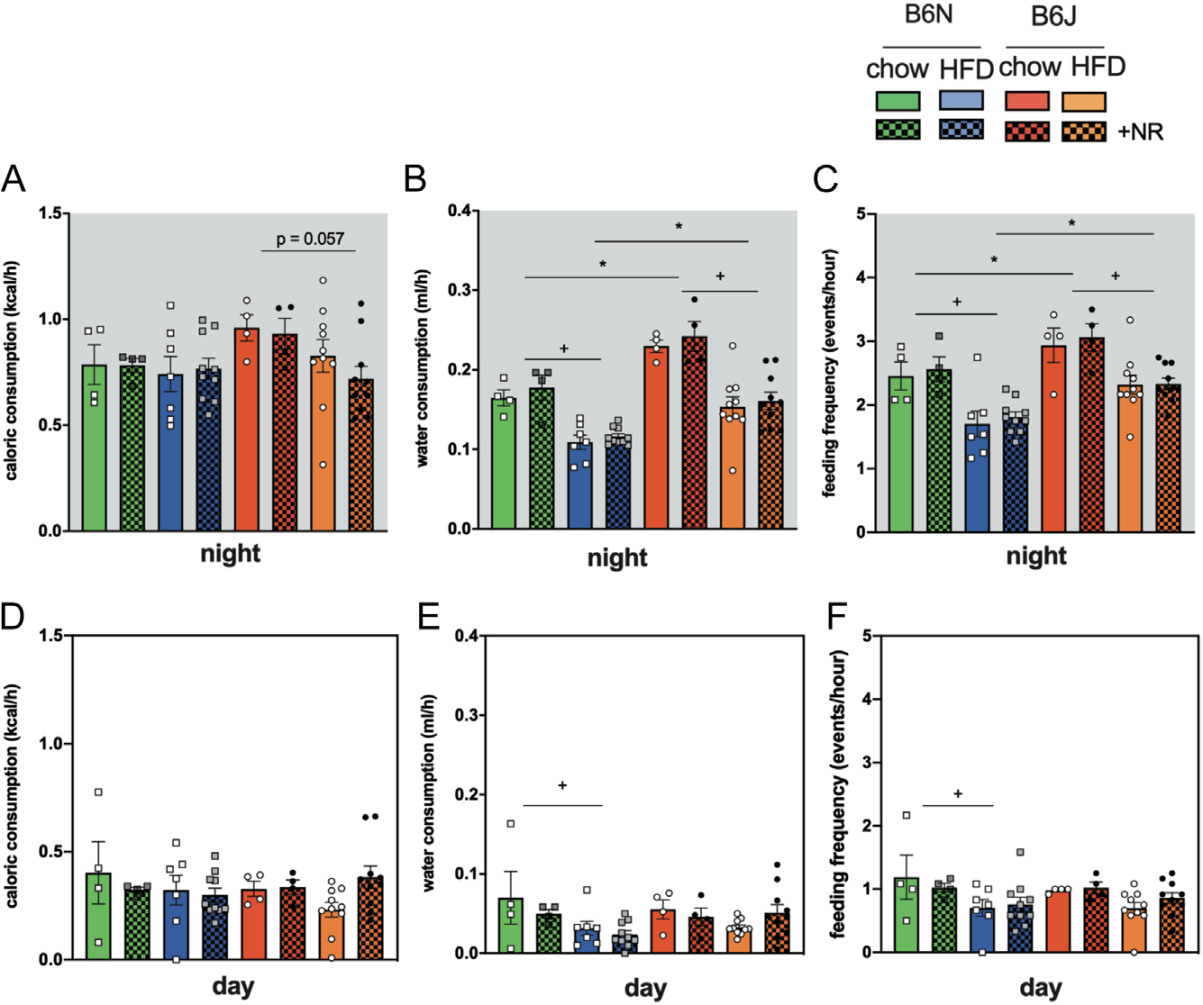
Water intake and feeding frequency are altered by NR supplementation in HFD-fed mice. Caloric consumption (A and C) and water intake (B and D) as well as feeding frequency (C and F) as a measure for activity were determined by indirect calorimetry in metabolic cages. Upper panel: night/active phase, lower panel: day/resting phase. Data are presented as mean ± s.e.m., *n = *4 for chow, *n = *7 for B6N HFD, *n = *10 for B6N HFD+NR, B6J HFD, and HFD+NR. Tests for statistical significance were done using ordinary two-way ANOVA with diet and mouse strain as factors followed by Tukey’s test for multiple comparisons. Data for NR vs no NR supplementation were analyzed together since no significant differences were found for NR vs no NR. ^+^*P  <* 0.05 for HFD vs chow, ^*^P  < 0.05 for B6J vs B6N.

Except for a borderline significantly higher caloric consumption in chow-fed vs HFD-fed B6J mice (Fig. 5A), there were no differences on either diet during night and day (Fig. 5A and D). In contrast, we found significant differences both in water intake (Fig. 5B) and feeding frequency (Fig. 5C) during the active phase and for B6N mice during the resting phase (Fig. 5E and F). Chow-fed mice of both strains had a higher water intake (Fig. 5B ^+^*P  <* 0.05 for HFD vs chow-fed) and were more active as indicated by feeding frequency (Fig. 5C, ^+^*P  <* 0.05 for HFD vs chow-fed). B6J mice drank significantly more water compared to B6N both on chow and on HFD during the night (Fig. 5B, ^*^P  < 0.05 for B6J vs B6N) and also were more active (Fig. 5C, ^+^*P  <* 0.05 for HFD vs chow-fed).

### NR supplementation induced increases in mitochondrial respiration in permeabilized soleus muscle fibers

**Figure 6:**
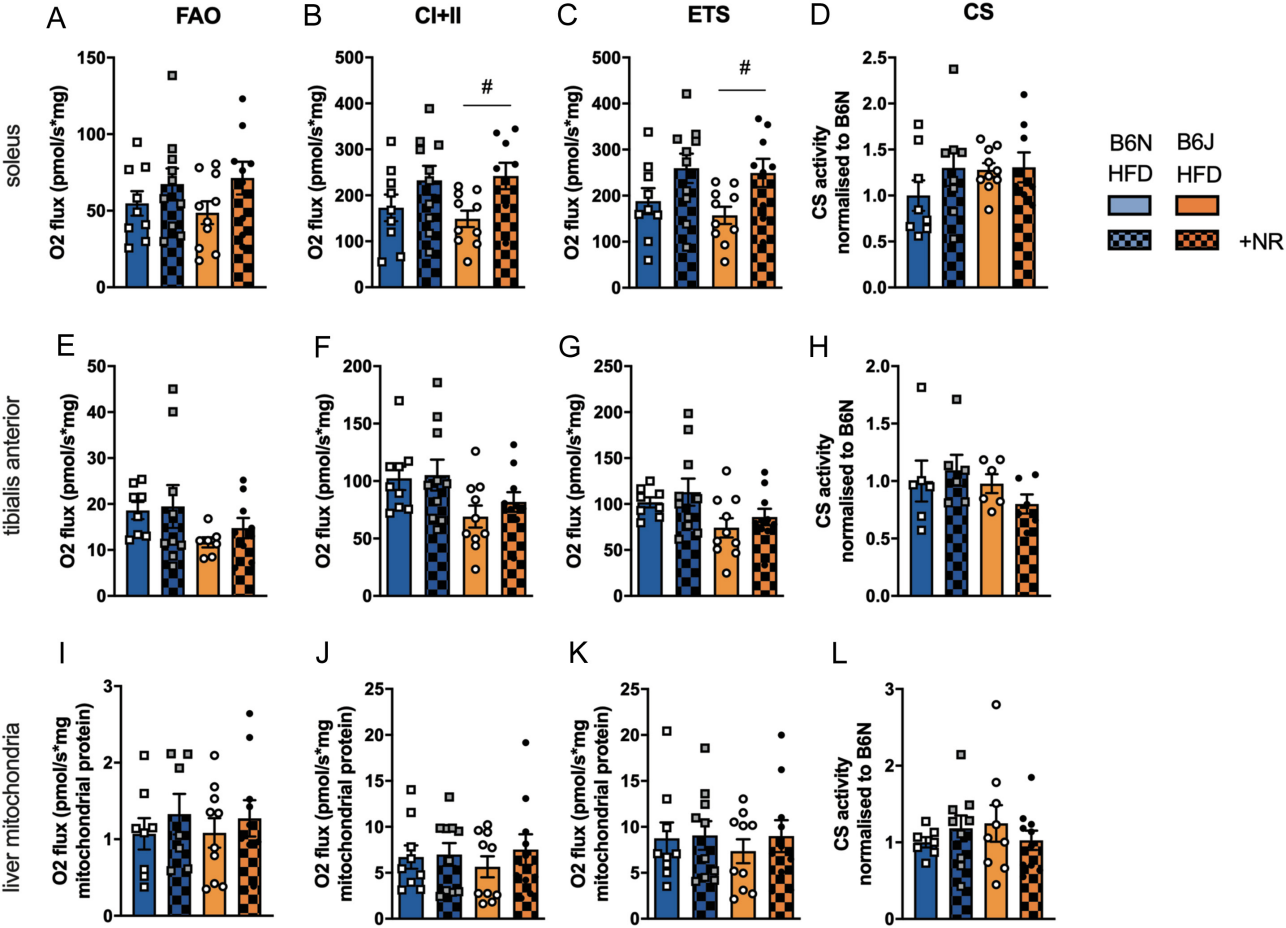
Mitochondrial respiration is higher in soleus muscle fibers from NR-supplemented compared to non-supplemented HFD-fed mice. Soleus and tibialis anterior permeabilized muscle fibers as well as liver mitochondria were subjected to high-resolution respirometry in the presence of ADP, *n = *9–10 per group. FAO, fatty acid (octanoyl-carnitine) oxidation, CI+II oxidation of octanoyl-carnitine, malate, and glutamate, ETS electron transport system maximum respiratory capacity induced by uncoupling of mitochondria with carbonyl cyanide-p-trifluoromethoxyphenylhydrazone (FCCP). CS, citrate synthase activity was measured enzymatically, *n = *6 per group. Data are presented as mean ± s.e.m. Tests for statistical significance were done using ordinary two-way ANOVA with NR supplementation and mouse strain as factors followed by Tukey’s correction for multiple comparisons. ^#^*P  <* 0.05 for NR vs no NR.

NR supplementation was previously shown to improve mitochondrial function in the liver and muscle of obese mice (Cantó *et al.* 2012). We measured respiration in the liver mitochondria and different permeabilized muscle fibers in mice after 8 weeks of HFD with and without NR supplementation. We found higher respiration in soleus muscle fibers of NR-supplemented mice for B6J mice (Fig. 6A, B and C), specifically when mitochondria were supplied with substrates for complexes I and II and ADP (Fig. 6B, ^#^*P  <* 0.05 for NR vs no NR) or when respiration was uncoupled (Fig. 6C, ^#^*P  <* 0.05 for NR vs no NR). Mitochondrial respiration of tibialis anterior (TA) muscle fibers (Fig. 6E, F and G) or liver mitochondria (Fig. 6I, J and K) was not significantly influenced by NR supplementation. Citrate synthase activity was not significantly different in any tissue (Fig. 6D, H and L), indicating that mitochondrial mass was not changed by NR supplementation.

## Discussion

We asked whether the metabolic responses to supplementation with NR could be influenced by the choice of mouse strain and compared C57B/B6J with C57B/B6N mice, both of which are established inbred mouse strains for the study of diet-induced obesity (Podrini *et al.* 2013). B6N mice differ from B6J in a number of single nucleotide polymorphisms that may influence the development of obesity, such as *Snap29* and *Aplp2* (Heiker *et al.* 2013, Smoczek *et al.* 2020). In addition, B6N, in contrast to B6J, is functional for nicotinamide nucleotide transhydrogenase (Nnt) (Mekada *et al.* 2009). This mitochondrial enzyme catalyzes the conversion of reduced NADH to reduced NADPH and, therefore, is crucial for mitochondrial redox balance and oxidative stress defense (Ronchi *et al.* 2013, 2016, Fisher-Wellman *et al.* 2015, Navarro *et al.* 2017), while Nnt’s role in insulin secretion is not yet clear (Toye *et al.* 2005, Freeman *et al.* 2006, Aston-Mourney *et al.* 2007, Fisher-Wellman *et al.* 2016). Numerous studies on repleting NAD by supplementation with NR showed beneficial effects on mitochondrial function and energy metabolism in mouse models of metabolic and mitochondrial disease (Cantó *et al.* 2012, Felici *et al.* 2015, Lee *et al.* 2015, Trammell *et al.* 2016, Zhou *et al.* 2016, Schöndorf *et al.* 2018, Crisol *et al.* 2020, Han *et al.* 2019). Male C57B/6J mice were used in most of these studies to model obesity, type 2 diabetes, and metabolic dysfunction.

We subjected both B6J and B6N mice to 8 weeks of HFD, which is a well-described regimen to induce a mild obesity phenotype and impaired glucose tolerance (de Wilde *et al.* 2009, Naznin *et al.* 2017, Zhou *et al.* 2017, Chang *et al.* 2018). Unexpectedly, B6J mice did not gain as much weight as B6N. This was reflected by a seemingly higher capacity of B6N mice to store fat both in adipose tissue depots and in the liver. Other studies examining HFD-induced obesity reported conflicting results regarding weight gain of B6J mice (Koza *et al.* 2006, Nicholson *et al.* 2010, Boulangé *et al.* 2013, Kahle *et al.* 2013, Fisher-Wellman *et al.* 2016). As expected and described before, we detected higher blood glucose levels during a glucose tolerance test in B6J and B6N mice after 8 weeks of HFD (Nicholson *et al.* 2010, Fontaine & Davis 2016) compared to chow.

In contrast to other studies (Cantó *et al.* 2012, Trammell *et al.* 2016), we did not detect a decrease in body weight, weight of fat depots, or improvement of glucose metabolism in NR-supplemented animals. Mice received 3.0 mg/mL NR in drinking water, which is equivalent to approximately 500 mg/kg/day and resulted in an approximate daily uptake of NR of 14–18 mg for chow and 7–10 mg for HFD-fed mice. NR concentration and mode of uptake were similar to other studies, which reported increased NAD, NADH, and ATP concentrations in the liver of NR-supplemented animals (Mukherjee *et al.* 2017, 2021). Using this dose, a beneficial effect on liver regeneration following partial hepatectomy was observed by Mukherjee *et al* (Mukherjee *et al.* 2017). To study the effects of NR supplementation in feed, a dose of 400–450 mg/kg/day was used in several mouse studies and beneficial effects on mitochondrial function, energy metabolism, muscle stem cell regeneration, and cardiac function were observed (Cantó *et al.* 2012, Gariani *et al.* 2016, Ryu *et al.* 2016, Diguet *et al.* 2018).

Since B6J mice on HFD drank more water than B6N, their exposure to NR was potentially higher. Similarly, chow-fed mice drank more water than HFD-fed mice of both strains. Our study is limited in that we could not measure NAD of B6N NR-supplemented in comparison to non-supplemented mice due to limited sample availability. When checking total NAD in the liver and cardiac muscle of B6J HFD-fed mice, we saw a significant increase. Another limitation of the study is the small number of mice on chow diet, which potentially led to false-negative results regarding the effect of NR supplementation on energy expenditure and RER.

In previous studies, NR supplementation was shown to be effective in mice with strong obesity or diabetic phenotype (Cantó *et al.* 2012, Trammell *et al.* 2016). Our mice were on a HFD for 8 weeks to model early phase weight gain and changes in glucose tolerance and energy metabolism (Rendina-Ruedy *et al.* 2015). The phenotypic changes, therefore, were slighter than for mice on a 21-week-HFD (Trammell *et al.* 2016) or on a high-fat high-sucrose diet (Cantó *et al.* 2012, Gariani *et al.* 2016). The validity of comparing results from this study of NR effects in mice with NR supplementation studies in humans is obviously limited. Similarly to our results though, NR supplementation in mildly obese or aged subjects did not result in substantial improvements in energy metabolism, had no effect on endocrine pancreatic function or on skeletal muscle mitochondrial function (Dollerup *et al.* 2018, 2019*a*,*b*, Martens *et al.* 2018, Elhassan *et al.* 2019).

Few animal studies also reported no benefits (Purhonen *et al.* 2018, Spaulding *et al.* 2019, Frederick *et al.* 2020) or even detrimental effects induced by NR supplementation, such as decreased exercise capacity and systemic oxidative stress in rats (Kourtzidis *et al.* 2016, 2018), decreased metabolic flexibility in mice (Shi *et al.* 2017) or glucose intolerance and white adipose tissue dysfunction in mice (Shi *et al.* 2019). In some of these studies, NR was used at lower doses as in our experiments. Specifically, Shi *et al.* used NR supplementation at 900 mg NR per kg diet, which corresponds to an NR dose of approx. 100 mg/kg/day and detected no beneficial effects on energy metabolism. Instead, compromised metabolic flexibility (the ability to rapidly switch metabolism between carbohydrate oxidation and fatty acid oxidation) was found by indirect calorimetry (Shi *et al.* 2017). At the ten-fold NR dose (9000 mg NR/kg diet), Shi *et al.* observed a decreased glucose clearance rate accompanied by an impaired insulin response along with the decreased metabolic flexibility. This observation was probably due to reduced insulin responsiveness and increased inflammation of epidydimal white adipose tissue in NR-supplemented compared to control mice. In contrast to our 8 week study, mice received a high dose of NR for 18 weeks (Shi *et al.* 2019).

When comparing mitochondrial respiration in supplemented vs non-supplemented mice on HFD, we detected increased ADP-dependent and uncoupled respiration in soleus muscle fibers from NR-supplemented to non-supplemented B6J mice, with a similar trend seen in B6N. NR did not influence the activity of citrate synthase as a measure for mitochondrial mass. In a previous study, soleus was shown to take up nicotinamide mononucleotide (NMN) and subsequently increase mitochondrial respiration (Mills *et al.* 2016). The response to HFD was shown to be fiber-type selective (Pinho *et al.* 2017, Leduc-Gaudet *et al.* 2018). Whether soleus fibers are more responsive to NR, take up more NR, or more sufficiently replete NAD when supplemented with NR should be determined in future studies.

In our hands, the beneficial effects of NR supplementation reported for HFD-induced obesity mouse models were not apparent. We only detected slight mouse-strain-dependent variation in the response to NR supplementation. In line with this, a recent study examining indirect calorimetry results from two large mouse projects revealed the institutional site as causing the biggest experimental variance (Corrigan *et al.* 2020). The prominent differences in metabolic phenotype in our study were seen between the two mouse strains, highlighting once more the importance of checking metabolic effects of nutritional supplements in multiple mouse strains.

## Supplementary Material

Supplementary Figure 1 A) Stability of nicotinamide riboside chloride (NR) was determined by incubation of NR in mouse drinking water at room temperature for 6 days followed by NMR quantification. After 6 days, the portion of NR was 95%. To check uptake of NR via the drinking water, total NAD concentrations in B) liver and C) cardiac muscle of HFD-fed B6J mice with and without NR administration (n = 4 in each group) were measured by an enzymatic cycling assay and normalised to protein concentrations. D) Genotyping results of mice used in this study. Mutant (743 bp) and wildtype (579 bp) nicotinamide nucelotide transhydrogenase were detected by PCR with specific primers according to Nicholson et al. 2010. E) Body weight time course of HFD and chow-fed mice (n = 10 for HFD B6N and B6J, n = 5 for chow diet B6N and B6J). Significant differences in weight were analyzed by three-way repeated analysis of variance followed by Tukey’s multiple comparisons test. Weight of F) inguinal and G) epidydimal fat depots was normalised to body weight of mice. (n = 8-10 for HFD-fed B6N, n = 7 for HFD-fed B6J, n= 5 for chow-fed B6N and B6J mice). Data are presented as mean±SEM * p<0.05 for B6J vs B6N, + p<0.05 for HFD vs chow.

Supplementary Figure 2: Analysis of covariance (ANCOVA) between metabolic flexibility as measured by the difference of RER between night and day (ΔRER) and body weight of A) chow-fed and B) HFD-fed mice. n = 8 for chow-fed B6N and B6J, n = 17 for HFD-fed B6N, n = 19 for HFD-fed B6J.

## Declaration of interest

The authors declare that there is no conflict of interest that could be perceived as prejudicing the impartiality of the research reported.

## Funding

This work was funded by the European Union, grant number 705869, a Wellcome Trust http://dx.doi.org/10.13039/100010269 Senior Fellowship, grant number GGL-104612/Z/14/Z, an Early Career Grant from the Society for Endocrinology http://dx.doi.org/10.13039/501100000382 and received support from the Research and Development Fund of the College of Medical and Dental Sciences, University of Birmingham, UK.

## Author contribution statement

Conceptualization, A G, D J H and G G L; methodology, A G, D M C, L O, R S F, C L D, S H, D P L, D N, D J H.; formal analysis, A G; investigation, D M C, L O, R S F, C L D, S H, D P L; C E B, S R H; resources, G G L; writing – original draft preparation, A G; writing – review and editing, A G; visualization, A G; supervision, D J H and G G L; project administration, A G and G G L; funding acquisition, A G and G G L.
