# Supplementary material for: Nicotinamide riboside has minimal impact on energy metabolism in mouse models of mild obesity
Source: J Endocrinol. 2021 Aug 9;251(1):111–23. doi: 10.1530/JOE-21-0123 (PMC8494379; doi:10.1530/JOE-21-0123)
Supplement: Supplementary Figure 1 A) Stability of nicotinamide riboside chloride (NR) was determined by incubation of NR in mouse drinking water at room temperature for 6 days followed by NMR quantification. After 6 days, the portion of NR was 95%. To check uptake of NR via the drinking water, total NAD concen [file supplementary_figure_1.pdf]

**Supplementary Figure 1**

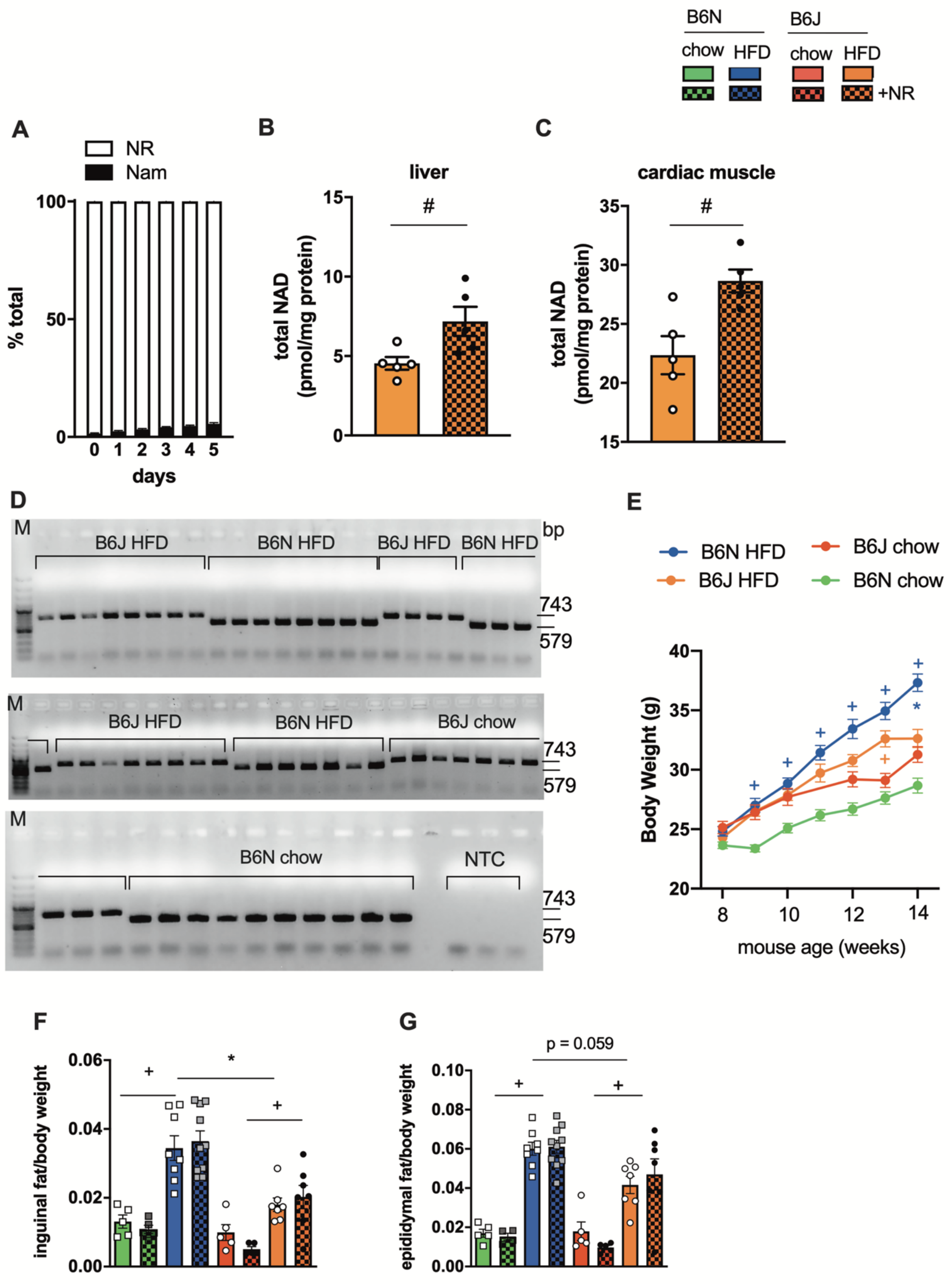


A) Stability of nicotinamide riboside chloride (NR) was determined by incubation of NR in mouse drinking water at room temperature for 6 days followed by NMR quantification. After 6 days, the portion of NR was 95%. To check uptake of NR via the drinking water, total NAD concentrations in B) liver and C) cardiac muscle of HFD-fed B6J mice with and without NR administration (n = 4 in each group) were measured by an enzymatic cycling assay and normalised to protein concentrations. D)

Genotyping results of mice used in this study. Mutant (743 bp) and wildtype (579 bp) nicotinamide nucelotide transhydrogenase were detected by PCR with specific primers according to Nicholson *et al.* 2010. E) Body weight time course of HFD and chow-fed mice (n = 10 for HFD B6N and B6J, n = 5 for chow diet B6N and B6J). Significant differences in weight were analyzed by three-way repeated analysis of variance followed by Tukey's multiple comparisons test. Weight of F) inguinal and G) epidydimal fat depots was normalised to body weight of mice. (n = 8-10 for HFD-fed B6N, n = 7 for HFD-fed B6J, n= 5 for chow-fed B6N and B6J mice).
Data are presented as mean±SEM * $p<0.05$ for B6J vs B6N, + $p<0.05$ for HFD vs chow.
