# Supplementary material for: Nicotinamide riboside has minimal impact on energy metabolism in mouse models of mild obesity
Source: J Endocrinol. 2021 Aug 9;251(1):111–23. doi: 10.1530/JOE-21-0123 (PMC8494379; doi:10.1530/JOE-21-0123)
Supplement: Supplementary Figure 2: Analysis of covariance (ANCOVA) between metabolic flexibility as measured by the difference of RER between night and day (ΔRER) and body weight of A) chow-fed and B) HFD-fed mice. n = 8 for chow-fed B6N and B6J, n = 17 for HFD-fed B6N, n = 19 for HFD-fed B6J. [file supplementary_figure_2.pdf]

Supplementary Figure 2

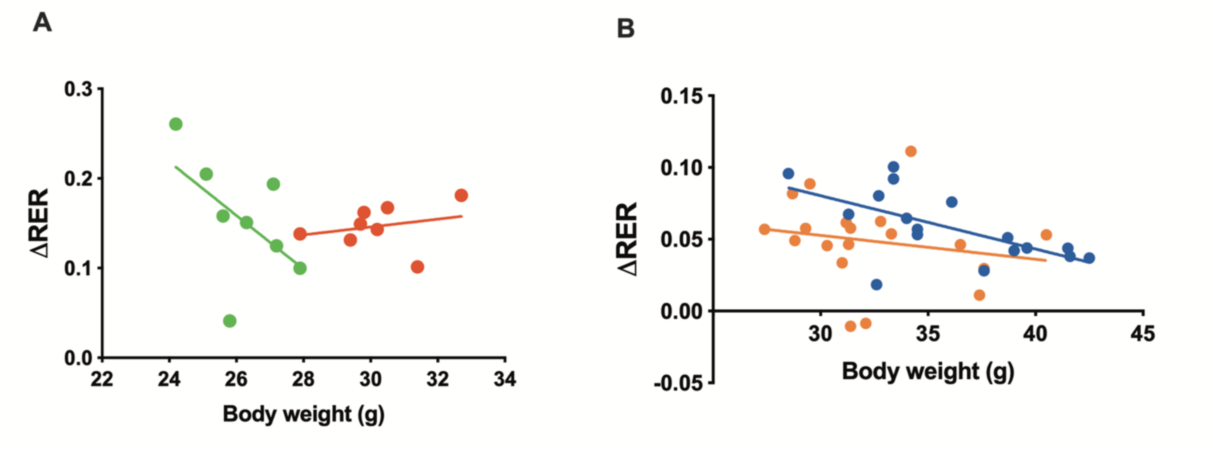

Supplementary Figure 2: Analysis of covariance (ANCOVA) between metabolic flexibility as measured by the difference of RER between night and day (ΔRER) and body weight of A) chow-fed and B) HFD-fed mice. n = 8 for chow-fed B6N and B6J, n = 17 for HFD-fed B6N, n = 19 for HFD-fed B6J
